# *Rubus caesius* leaves and stems in antiaging skin care products: antityrosinase, anticollagenase, antimicrobial activity, and transdermal distribution of main phenolic acids

**DOI:** 10.1080/13880209.2025.2576002

**Published:** 2025-11-03

**Authors:** Anna Hering, Anna Nowak, Anna Muzykiewicz-Szymańska, Rafał Tomasz Hałasa, Łukasz Kucharski, Paula Ossowicz-Rupniewska, Alina Kastsevich, Yahor Ivashchanka, Justyna Stefanowicz-Hajduk

**Affiliations:** a Department of Biology and Pharmaceutical Botany, Medical University of Gdańsk, Gdańsk, Poland; b Department of Cosmetic and Pharmaceutical Chemistry, Pomeranian Medical University in Szczecin, Szczecin, Poland; c Department of Pharmaceutical Microbiology, Medical University of Gdańsk, Gdańsk, Poland; d Department of Organic Chemical Technology and Polymer Materials, West Pomeranian University of Technology, Szczecin, Poland; e Department of Pharmacy, Medical University of Gdańsk, Gdańsk, Poland

**Keywords:** European dewberry, gallic acid, hydrogel, collagen, melanin, skin absorption

## Abstract

**Context:**

The skin is the largest organ of the body, and its proper care significantly influences the well-being of the entire organism. Therefore, the ingredients of cosmetics and dermocosmetics should inhibit processes leading to inflammation and degradation of skin macromolecules.

**Objective:**

To select the most promising *Rubus caesius* L. extract for use in cosmetic and dermocosmetic applications.

**Materials and methods:**

Water and ethanol extracts from leaves and stems of *Rubus caesius* L. (European dewberry, Rosaceae) were tested for their antioxidant properties, protective effects against pathogenic bacterial strains and the influence on tyrosinase and collagenase activity. The most biologically active extracts were selected and analyzed using the HPLC method to estimate the content of major phenolic acids and their ability to penetrate into and through porcine skin from hydrogels.

**Results:**

Ethanol extracts from *Rubus caesius* L. demonstrated significant biological activity, particularly in scavenging the ABTS radical and inhibiting tyrosinase and collagenase activity. Furthermore, ethanol extracts were effective against pathogenic bacteria, but not against commensal skin microbiota. Ethanol extracts from leaves and stems were rich in phenolic acids. The permeation experiment through porcine skin from hydrogels revealed that gallic acid and neochlorogenic acid from ethanol leaves extract exhibited the highest permeation capability.

**Discussion and conclusion:**

The ethanol extract demonstrated substantial activity in protecting the skin against pathogens, oxidative stress, and macromolecular degradation. The presence and transdermal permeability of phenolic acids were also confirmed. These findings highlight the high potential of *Rubus caesius* leaf ethanol extract for inclusion in cosmetic and dermocosmetic formulations.

## Introduction

In recent years, there has been a growing interest in the search for safe ingredients for dermatological and cosmetic preparations. Additives of natural origin, containing biologically active molecules, exhibit multiple effects on the skin and subcutaneous layers (Zagórska-Dziok et al. 2021a; Gonçalves and Gaivão 2023). The utility of mushroom extracts and the benefits of thermal waters for maintaining skin health have been known for centuries (Wu et al. 2016; Figueiredo et al. 2023). In particular, thermal waters are considered beneficial cosmetic ingredients due to their high mineral content (Polefka et al. 2012; Figueiredo et al. 2023; Mourelle et al. 2023). However, the vast plant kingdom offers the cosmetics industry a wide range of biologically active compounds with multifunctional effects.

Therefore, increasing attention is being paid to the use of plant extracts as sources of valuable secondary metabolites for dermatological applications. Antioxidant and antibacterial properties of such extracts are often combined with anti-inflammatory effect (Coyago-Cruz et al. 2024). A highly sought-after property is the inhibition of enzymes responsible for the degradation of macromolecules in the extracellular matrix (ECM). Plant extracts rich in biologically active molecules can effectively protect the skin and its structural macromolecules from external degradation (Fibrich et al. 2019; Nowak et al. 2021a). The skin is the organ most exposed to external factors, and its natural defenses weaken over time. Oxidative stress caused by smoking, environmental pollution, and UV radiation leads to accelerated aging processes. Reactive oxygen species (ROS) are responsible for activation of collagenase, elastase, and hyaluronidase, causing degradation of collagen, elastin and leading to skin dehydratation and saging (Rittie and Fisher 2002; Naylor et al. 2011). ROS and UV radiation are the most important factors stimulating tyrosinase activity, the key enzyme in melanin formation and generation of inflammation process that cause the release of reactive quinones (Ito et al. 2020). The destruction of collagen structures and disruption of melanin production result in deeper wrinkles and increased pigmentation (brown spots). Long-term, severe oxidative stress in the skin ultimately leads to irreversible pathogenic changes, including laxity, leathery appearance, solar elastosis, and even skin cancer (Rittie and Fisher 2002; Naylor et al. 2011; Ito et al. 2020; Nisa et al. 2024).

Another important factor for proper skin function is microbiome. It contains up to one million different species of viruses, fungi and bacteria (Chen and Tsao 2013, Lee et al. 2021). Disturbances in the skin microbiome’s balance may contribute to the development of various pathological conditions, including aging (Prescott et al. 2017, Li et al. 2020, Lee et al. 2021). Studies have shown that the skin of older people has a greater microbial diversity than that of younger individuals, which may result in higher susceptibility of older people’s skin to pathogen invasion. It has been noted that during adolescence, the density of lipophilic bacteria on the skin is getting higher with increasing sebum levels; unfortunately, it is lower in the skin of older people (Jugé et al. 2018). It was indicated that changes in the composition of the skin microbiome can influence many pathways occurring in the skin (e.g., MAPK signaling, glutathione metabolism, pantothenate and coenzyme A biosynthesis) that potentially influenced skin aging. This suggests that the skin microbiome may play a key role in skin aging by regulating the immune response, resistance to ultraviolet radiation, and the biosynthesis and metabolism of age-related substances (Li et al. 2020, Lee et al. 2021).

*Rubus caesius* L. (European dewberry, Rosaceae) is a small shrub commonly found throughout most of Europe and Eastern to Central Asia. In ethnomedicine, its various parts were utilized for medicinal purposes and were used both orally in the form of infusions and externally as ointments or poultices applied to the skin (Xiong et al. 2024). However, the qualitative and quantitative composition of active ingredients may vary significantly between different parts of *Rubus caesius* and between related species like *Rubus idaeus* (Hering et al. 2022; Kotuła et al. 2022).

European dewberry is mostly known for its fruits. Their sweet and sour taste, combined with a high content of polyphenolic and anthocyanin compounds, makes them a popular addition to the summer diet, especially in Northern Europe (Svanberg and Ståhlberg 2021). However, the fruits are only available seasonally, whereas the leaves persist from early spring until the first frosts. The leaves and stems analyzed in this study were collected before the flowering phase, during a period of vigorous growth and active biosynthesis of bioactive compounds. Leaves and stems of *Rubus caesius* are rich in flavonoids and may be considered effective ingredients for cosmetic and dermocosmetic formulations (Hering et al. 2022).

This study aimed to identify the most biologically active extract among the ethanol and water extracts of *Rubus caesius* leaves and stems harvested in spring. The selected extract should ideally demonstrate properties relevant to protecting the skin and its macromolecules from degradation and microbial infections. Additionally, for the first time, the composition of phenolic acids and their ability to penetrate into and through the skin from hydrogel formulations of *Rubus caesius* extracts were analyzed.

## Materials and methods

### Chemicals

Collagenase from *Clostridium histolyticum* (C5138), tyrosinase (tyrosinase from mushroom; T3824), tricine buffer (39468-M), N-[3-(2-Furyl)acryloyl]-Leu-Gly-Pro-Ala (FALGPA, F5135), 3-(3,4-Dihydroxyphenyl)-L-alanine (L-DOPA, 333786), phosphate buffer (0.175 mM, pH 6.8, P5244), 2,2′-azinobis (3-ethylbenzothiazoline-6-sulfonic acid diammonium salt (ABTS, 194434), potassium persulfate (216224), dimethyl sulfoxide (DMSO, 472301), kojic acid (K3125), oleanolic acid (O5504), ascorbic acid (1043003), protocatechuic acid (PHL89766), chlorogenic acid (PHR2202), caffeic acid (205546), ampicillin (PHR2838) and amphotericin (A2942) were purchased from Sigma Aldrich (Steinheim am Albuch, Germany); TRIS-HCl (1185-53-1), HCl (327-97-9), NaCl (7647-14-5), CaCl_2_ (10043-52-4), acetic acid (20123.363), sodium acetate (127-09-3) were sourced from Avantor Performance Materials Poland S.A. (Gliwice, Poland). Gallic acid (G7384), 4-hydroxybenzoic acid (H20059), 3-hydroxybenzoic acid (H20008), hydroxyethylcellulose (09368) and acetonitrile for HPLC (34851) were from Merck (Darmstadt, Germany). Acetic acid (99.5%, 42711), ethanol (37673), methanol (47813), and acetone (30113) were from Chempur (Piekary Śląskie, Poland). Propylene glycol (PA-04-D4283) was from Pol-Aura (Morąg, Poland). All reagents used were of analytical grade.

### Plant material

Plant material was collected before the flowering period from the cultivation in the experimental garden of medicinal plants belonging to the Department of Biology and Pharmaceutical Botany, Medical University of Gdańsk, Gdańsk, Poland (GPS 54.3823, 18.6239, May 2025). The botanical identification of *Rubus caesius* L. was made by Piotr Kosiński Ph.D. in 2021 (GPS 54.4195, 18.4416), the voucher specimen was deposited in the Herbarium of Medical University of Gdańsk, Poland (GDMA Herbarium No. 4636, 15.06.2021).

The plant material (leaves and stems were processed separately) was dried at room temperature in a well-ventilated area until a constant weight was achieved. Then, 2.5 g of dried leaves or stems were extracted with 100 mL of 70% (*v/v*) ethanol or distilled water for 60 min in an ultrasonic bath (Polsonic Sonic 5, Poland) operating at a frequency of 40 kHz. After filtration, the extracts were evaporated under reduced pressure at 40 °C (evaporator BUCHI Rotavapor R-200), lyophilized (Christ Alpha 1-2 LDplus) and divided into two portions. One portion was used for HPLC analysis, while the other was stored in the dark at 4 °C until further analysis. Prepared extracts: water extracts from leaves (L˗W) and stems (S˗W), ethanol extracts from leaves (L˗E) and stems (S˗E).

### Antioxidant tests

Water and ethanol extracts from *Rubus caesius* leaves and stems were freshly prepared by dissolving the dry extract in water. The samples were tested at concentrations ranging from 1 to 1000 µg/mL. Ascorbic acid was used as the reference standard in both assays. All measurements were performed using a 96-well microplate reader (Epoch, BioTek System, Winooski, VT, USA). IC_50_ values for each extract were calculated using the GraphPad Prism 9 software (version 9.0.0, GraphPad Software, San Diego, CA, USA). Each test was performed in triplicate and repeated three times (*n* = 9).

#### ABTS radical assay

The antioxidant activity of the *Rubus caesius* extracts was determined using ABTS radical scavenging method as described by Thring et al. (2009), with minor modifications according to Hering et al. (2025). The reaction mixture containing extracts or standard were incubated in the dark for 20 min at 30 °C. After incubation, absorbance was measured at 750 nm. The negative control sample consisted of the reaction mixture and water instead of extract or standard.

#### Molybdenum reduction assay

The total reducing power of water and ethanol extracts from *Rubus caesius* was estimated according to the phosphomolybdenum method described by Prieto et al. (1999), with modifications by Hering et al. (2025). The samples (extract or standard) were mixed with the reagent solution and incubated at 90 °C for 90 min. After cooling, 250 µL of each reaction mixture was transferred to a 96-well microplate reader (Epoch, BioTek System, Winooski, VT, USA), and absorbance was measured at 700 nm. The negative control consisted of the reaction mixture and water.

### Enzymes inhibition

Preparation of the extracts and calculation of IC_50_ values were carried out as described in the *Antioxidant tests* section. The effect of *Rubus caesius* extracts on collagenase and tyrosinase activity was estimated using the spectrophotometric method in a 96-well microplate reader (Epoch, BioTek System, Winooski, VT, USA).

The addition of the substrate started the reaction. Changes in absorbance were monitored every 20 s for a total of 20 min. The blank sample contained buffer, substrate, and the appropriate concentration of extract or standard. The negative control sample contained water instead of extract or standard. All tests were performed in triplicate and repeated three times (*n* = 9).

#### Tyrosinase inhibition assay

The tyrosinase inhibition assay was performed according to the method described by Yagi et al. (1987), with modifications by Hering et al. (2023). L-DOPA was used as the substrate, and kojic acid served as the positive control. The reaction mixture consisted of phosphate buffer (0.175 mM, pH 6.8), 15 µL of tyrosinase (120 U), and serial dilutions of *Rubus caesius* extracts (0–400 µg/mL). The solutions were pre-incubated in the dark for 15 min. The reaction was initiated by adding 10 mM L-DOPA, and absorbance was measured at 475 nm to monitor product formation.

#### Collagenase inhibition assay

The effect of *Rubus caesius* extracts on collagenase activity was assessed based on the method described by Thring et al. (2009), with modifications introduced by Hering et al. (2023). FALGPA was used as the substrate, and oleanolic acid served as the positive control. Reaction mixtures containing the extract (0–400 µg/mL) were pre-incubated for 15 min. The reaction was initiated by the addition of 0.8 mM FALGPA, and absorbance was continuously measured at 335 nm. The reaction buffer was composed of tricine (50 mM, pH 7.5) with 400 mM NaCl and 10 mM CaCl_2_.

### Antimicrobial study

#### Materials

Brain-heart infusion broth (BHI, Becton Dickinson) supplemented with 10% bovine serum was used for the cultivation of *Streptococcus. Streptococcus* β-hemolytic group G and *Cutibacterium acnes* (formerly *Propionibacterium acnes*, ATCC6919) were incubated anaerobically using GENbag anaerobe (BioMerieux) at 37 °C for 48 h. *Staphylococcus epidermidis* (ATCC14990) and *Bacillus subtilis* (ATCC6633) were grown in Mueller˗Hinton broth (MH cation-adjusted, Becton Dickinson) under aerobic conditions at 37 °C for 48 h. *Corynebacterium diphtheriae* ZMF was cultured in Brain-heart infusion broth (BHI, Becton Dickinson) supplemented with 10% bovine serum under aerobic conditions at 37 °C for 72 h. Fungal strains-*Candida albicans* (ATCC10231)*, Candida albicans (*ATCC26790)*, Candida glabrata (*ATCC200)*, Candida krusei (*ATCC6258)*, Candida parapsilosis (*ATCC22019) were grown in Sabourauda broth (SB, Becton Dickinson), under aerobic conditions at 37 °C for 72 h. For post-incubation viability assessment, BHI blood agar plates, MH agar plates, and Sabouraud agar plates were used.

#### Antibacterial assay

Active cultures were prepared by transferring cells from stock cultures into appropriate broth, as described above. These cultures were incubated without agitation for 24 or 48 h at 37 °C. Cultures were then diluted in the same broth to achieve an optical density corresponding to approximately 10^6^ colony-forming units per milliliter (CFU/mL) for all bacteria strains except *C. acnes* and *C. diphtheriae* ZMF. For these two species, inocula were prepared from colonies grown on BHI blood agar plates incubated for 48 h under anaerobic (*C. acnes*) or aerobic (*C. diphtheria)* conditions, adjusted to ∼ 10^6^ CFU/mL (Hałasa et al. 2014).

The minimum inhibitory concentration (MIC) was determined using the broth microdilution method in 96-well plates. Each well was filled with 100 µL of broth. Dried test samples were dissolved in water or dimethyl sulfoxide (DMSO) to a stock concentration of approximately 100 mg/mL. These solutions were serially diluted and added to the first well of each row. Serial twofold dilutions were performed by transferring 100 µL from well to well up to the twelfth well, from which 100 µL was discarded. Final concentrations of the extracts ranged from 10 to 0.005 mg/mL. Ampicillin served as the reference standard, with concentrations ranging from 128 to 0.0625 µg/mL. Plates were incubated under the appropriate conditions at 37 °C for 48 (*Clostridium* sp. was incubated anaerobically). MIC values were determined by visual observation and defined as the lowest extract concentration that prevented visible microbial growth. To determine the minimum bactericidal concentration (MBC), 100 µL from wells showing no visible growth were plated on agar media. After 48 h of incubation, plates were assessed for colony formation. The MBC was defined as the lowest concentration of extracts required to completely eliminate viable microorganisms (Hałasa et al. 2014).

### HPLC analysis

The concentration of target compounds in the ethanol extracts of *Rubus caesius* was determined using high-performance liquid chromatography with UV detection (HPLC-UV), utilizing a Knauer system (Berlin, Germany). Separation was achieved on C18 column (Eurospher 100, 125 mm x 4 mm, 5 μm particle size). The isocratic mobile phase was used, isoconsisted of 1% acetic acid and MeOH (93:7, *v*/*v*), delivered at a flow rate of 1 mL/min. A 20 µL sample was injected for each analysis.

Compounds were identified using reference standards. The calibration curves yielded the following correlation coefficients (R^2^) and retention times (t_R_): gallic acid (y = 300074x − 1.1923, R^2^ = 0.9999, t_R_ = 6.455 min); protocatechuic acid (y = 20740x − 0.5806, R^2^ = 0.9999, t_R_ = 14.525 min); caffeic acid (y = 20466x − 0.0212, R^2^ = 0.9999, t_R_ = 16.673 min); neochlorogenic acid (y = 31388x + 0.2252, R^2^ = 0.9997, t_R_ = 21.027 min); coumaric acid (y = 39902x − 2.2879, R^2^ = 0.9998, t_R_ = 28.209 min); vanillic acid (y = 16798x + 0.2776, R^2^ = 1.0000, t_R_ = 46.442 min); and chlorogenic acid (*y* = 8327.5x + 0.057, R^2^ = 0.9998, t_R_ = 68.027). All samples were analyzed in triplicate. Results are reported as mean ± standard deviation (SD).

### Hydrogel preparations

Hydrogels were prepared following a modified method by Zagórska-Dziok et al. (2021a). A total of 0.25 g of the dried ethanol extract (from leaves or stems, separately) was dissolved in 0.5 g of propylene glycol and subsequently dispersed in 4.25 g of 1% hydroxyethylcellulose (HEC)-based gel. The gel was prepared by adding HEC to distilled water and stirring with a magnetic stirrer (Chemland MS-H280-Pro, Poland) at 40 °C and 250 rpm.

The final concentration of the dry extract in the hydrogel was 5%. Two hydrogels were obtained, designated HL-E and HS-E, containing ethanol extracts from *Rubus caesius* leaves and stems, respectively.

#### Stability of hydrogels

The stability of the hydrogels was assessed using a modified protocol based on Muthachan and Tewtrakul (2019). One gram of each hydrogel was centrifuged (MPW-223e, Mechanika Precyzyjna, Warsaw, Poland) at 4000 rpm for 10 min at 25 °C to evaluate phase separation.

Additionally, a heating-cooling cycle test was performed: samples were incubated at 45 °C for 48 h (Drying Oven, DHG-9075A), followed by 48 h at 4 °C. This cycle was repeated six times. All *Rubus caesius* extract-containing hydrogels maintained their consistency and appearance throughout the test.

### Skin penetration studies

Permeation experiments were conducted using Franz diffusion cells (Phoenix DB-6, ABL&E-JASCO, Wien, Austria), with a donor chamber area of 1 cm^2^ and 10 mL acceptor chamber filled with phosphate-buffered saline (PBS, pH 7.4). The system was maintained at 37.0 ± 0.5 °C, and the acceptor solutions were continuously stirred.

Porcine skin, which resembles human skin in structure and properties (Khiao et al. 2019; Jacobi et al. 2007), was obtained from a local slaughterhouse. Skin samples were wrapped in aluminum foil, frozen at −20 °C for no longer than three months. This frozen storage time was safe to keep skin barrier properties. On the day of the experiment, the skin samples were slowly thawed at room temperature for 30 min and were hydrated with PBS buffer pH 7.4 (Kuntsche et al. 2008; Simon et al. 2016; Haq and Michniak-Kohn 2018). Only undamaged skin samples with impedance values above 3 kΩ were used, as verified with an LCR meter 4080 (Voltcraft LCR 4080, Conrad Electronic, Germany), which was operated in parallel mode at an alternating frequency of 120 Hz (error at kΩ values <0.5%), operating at 120 Hz (error margin <0.5%). Skin impedance was measured by immersing the probe tips in the donor and acceptor chambers filled with PBS.

One gram of each hydrogel was applied to the donor chamber. All chambers were sealed with plastic stoppers to minimize evaporation. The experiment lasted 24 h. At specific time points (1, 2, 3, 5, 8, and 24 h), 0.3 mL of the acceptor solution was collected and replaced with fresh PBS (Kopečná et al. 2017; Makuch et al. 2020). Phenolic acid concentrations were determined by HPLC.

Cumulative mass (µg) and permeation parameters were calculated, including steady-state flux (J_SS_), permeability coefficient (K_P_), time required to reach steady-state permeation (lag time, L_T_), the diffusion coefficient (D), skin partition coefficient (K_m_), and the percentage of the applied dose after 24 h (Q_%24 h_). J-shaped profiles and the following equations were used:

(1) $$A=J_{\text{SS}}\left(t-L_{T}\right)$$

where A is the cumulative amount permeated (μg·cm^−2^) of tested phenolic acids permeating into the receptor compartment; J_SS_ is the steady-state flux (μg·cm^−2^·h^−1^); t is the time (h), and L_T_ is the lag time (h).

The steady-state flux was estimated from the slope of the linear portion of the plot of cumulative mass in the acceptor phase over time. The lag time (L_T_) was determined from the x-intercept of the linear portion of the plot of cumulative mass in the acceptor phase over time and was used to calculate the diffusion coefficient (K_P_) as follows:

(2) $$K_{P}=J_{\text{SS}}/C$$

where C is the concentration in the donor phase.

### Skin accumulation studies

Accumulation of the phenolic acid in the skin was determined using a modified protocol based on Ossowicz-Rupniewska et al. (2022). After 24 h of permeation, skin samples were removed, rinsed with PBS (pH 7.4), and cut around the diffusion area (1 cm^−2^) (Kopečná et al. 2017). The samples were air-dried and then minced, placed in 2 mL of methanol, and incubated at 4 °C for 24 h. Following incubation, samples were homogenized (IKA^®^T18 digital ULTRA TURRAX, Germany) for 3 min and centrifuged at 3500 rpm for 5 min. The resulting supernatants were analyzed by HPLC spectrophotometry, with methanol used as a blank. Accumulated phenolic acids were expressed as amount (µg) per gram of skin tissue (µg·g skin^−1^).

### Statistical analysis

Data are expressed as mean ± standard deviation (SD). One-way analysis of variance (ANOVA) followed by Tukey’s post hoc test was used to evaluate statistical differences between hydrogel formulations. A significance level of α < 0.05 was adopted. Statistical analyses were performed using Statistica 13 PL software (StatSoft, Cracow, Poland).

## Results

### Antioxidant tests

Antioxidant tests were performed in order to estimate the quality of the plant material as a potential source of antiradical and reduction agents. Extracts from *Rubus caesius* were subjected to ABTS radical scavenging ability and molybdenum reduction properties. The results expressed as IC_50_ value with comparison to the standard-ascorbic acid are presented in Table 1. Extracts from *Rubus caesius* indicated high capability to reduce ABTS radical, especially extracts from leaves, which exhibited IC_50_ similar to ascorbic acid, while ethanol extract was even stronger than the standard substance (IC_50_=15.04 ± 0.51; 11.66 ± 0.18 and 14.77 ± 0.5 µg/mL, for L-W, L-E, and ascorbic acid, respectively). Extracts from stems were also very active against ABTS radical and similar to the leaves. Ethanol extract from stems was stronger than water extract (IC_50_= 22.23 ± 0.16 and 35.63 ± 0.34 µg/mL, for S-E and S-W, respectively).

**Table 1.** Antiradical and molybdenum reduction properties of water (W) and ethanol (E) extracts from leaves (L) and stems (S) of *Rubus caesius* [µg/mL].

	L-W	S-W	L-E	S-E	Ascorbic acid
ABTS	15.04 ± 0.51	35.63 ± 0.34*	11.66 ± 0.18*	22.23 ± 0.16*	14.77 ± 0.5
Molybdenum reduction	454.24 ± 1.58*	569.08 ± 0.7*	140.35 ± 1.41*	99.09 ± 0.39*	78.25 ± 1.25

Ascorbic acid was used as a standard.

Statistically significant differences between results and the control are marked with an asterisk ‘*’, t-Student’s test, p < 0.05

Ethanol extracts exhibited promising capability to reduce molybdenum (IC_50_=99.09 ± 0.39; 140.35 ± 1.41; 78.25 ± 1.25 µg/mL, for S-E, L-E, and ascorbic acid, respectively), while water extracts reduced molybdenum only in average power (IC_50_=454.24 ± 1.58 and 569.08 ± 0.7 µg/mL, for L-W and S-W, respectively).

Dose-dependent capability to scavenge free radicals and reduce molybdenum was observed for all obtained from spring season *Rubus caesius*’ extracts. The results indicated that ethanol extracts are more capable to protect macromolecules from oxidative stress and can be utilized as highly probable sources of biologically active chemicals. As a result, they may constitute a positive impact on the functioning and condition of the skin, especially on reduction of inflammation and formation of reactive oxygen species (ROS) (Joun et al. 2024).

### Enzymes inhibition

In order to determine the antiaging effect of *Rubus caesius* extracts on skin, two enzymes connected with some of the first signs of skin aging: tyrosinase and collagenase, were tested. Excessive activity of tyrosinase leads to the formation of uneven skin tone, associated with a disturbance in melanin synthesis, while in the case of high activity of collagenase, the destruction of the collagen skeleton and skin sagging are observed (Naylor et al. 2011; Niu and Aisa 2017; Reilly and Lozano 2021).

**Figure 1.**
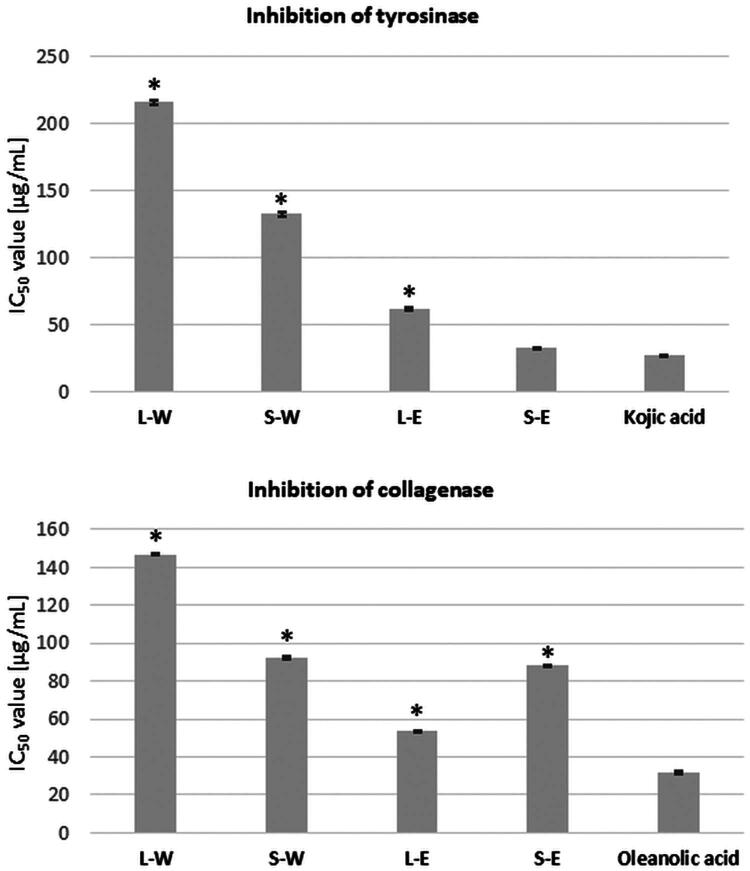
IC_50_ values of tyrosinase and collagenase inhibition by *Rubus caesius* water and ethanol extracts obtained from leaves (L-W, L-E) and stems (S-W, S-E). Statistically significant differences between results and the control (kojic acid or oleanolic acid, respectively) are marked with an asterisk ‘*’, t-Student’s test, p < 0.05.

The evaluation of the effect of *Rubus caesius* extracts on tyrosinase and collagenase activity was performed based on the spectrophotometric methods described by Yagi et al. (1987) and Thring et al. (2009) with modifications introduced by the authors (Hering et al. 2023). The results expressed as IC_50_ values are presented in Figure 1.

Studies indicated that all tested extracts exhibited capability to dose-dependent inhibition of tyrosinase and collagenase, although resulted influence on enzymes was weaker than standards (kojic acid and oleanolic acid for tyrosinase and collagenase inhibition, respectively).

The analysis of tyrosinase inhibition revealed that among the tested probes, ethanol extracts indicated a higher inhibitory effect on tyrosinase activity than water extracts (Figure 1). In addition, extracts prepared from the stems have a stronger antityrosinase effect than extracts obtained from the leaves.

The strongest, and similar to kojic acid, antityrosinase activity was presented by ethanol extract obtained from stems (IC_50_=26.79 ± 0.15 and 32.39 ± 0.36 µg/mL, for kojic acid and S-E, respectively). Twice weaker was the ethanol extract from leaves (IC_50_=61.61 ± 1.44 µg/mL), while water extracts presented significantly lower IC_50_ values against the enzyme (IC_50_=215.61 ± 1.2 and 132.52 ± 1.61 µg/mL for L-W and S-W extracts, respectively). Despite weaker IC_50_ values compared to kojic acid, it can be considered that ethanol extracts obtained from *Rubus caesius* leaves and stems show strong tyrosinase-inhibiting activity. Additionally, it should be emphasized that water extracts, in spite of lower antityrosinase activity, can also be considered highly active against tyrosinase and melanin malfunction.

The analysis of collagenase inhibition (Figure 1) indicated that the strongest influence on the enzyme activity was exhibited by ethanol extract from leaves, with IC_50_ value twice weaker than the standard (IC_50_=53.61 ± 0.14 and 31.72 ± 0.65 µg/mL, for L-E and oleanolic acid, respectively). Extracts from stems presented similar potential to inhibit collagenase, with slightly better results for ethanol extract (IC_50_=88.01 ± 0.73 and 92.1 ± 0.65 µg/mL, for S-E and S-W, respectively). Water extract from leaves was also effective in reducing collagenase activity, although it was more than four times weaker than the standard (IC_50_=146.97 ± 0.4 µg/mL).

Confirmed capability of the tested extracts from *Rubus caesius* to inhibit tyrosinase and collagenase *in vitro* suggests their high antiaging effectiveness in dermal application. Ethanol extracts deserve special attention due to their IC_50_ values that are more similar to the standard substances than values relating to water extracts.

### Antimicrobial study

For the antimicrobial study, the extracts from *Rubus caesius* were tested against bacterial strains that can significantly change the proper functioning of individual skin layers. As a result of bacterial potential activity, inflammation, purulent conditions, or boils may occur. Poorly selected antibiotics, their unsystematic use can cause antibiotic resistance and deepen inflammation. The use of plant extracts with antibacterial effects in cosmetic products significantly improves the condition of the skin, not only during infection but can also limits the settlement of pathogenic bacteria on its surface (Popova et al. 2022; Zouine et al. 2024).

The Rosaceae family is known for its antibacterial activity. It has been proven that individual morphological parts of plants belonging to this family exhibit different antibacterial effects (Nowak et al. 2014; Ispiryan et al. 2024). As the most active against bacterial strains are fruits and inflorescence (Nowak et al. 2014; Gomathi et al. 2024; Ispiryan et al. 2024), however, their harvesting is limited by the growing season. Tests were performed on water and ethanol extracts from leaves and stems from *Rubus caesius* (Tables 2 and 3). In the antimicrobial and antifungal studies, extracts were dissolved in water and in DMSO to assess the potential effect of compounds soluble in organic and polar solvents.

**Table 2.** Antibactieral properties of water (W) and ethanol (E) extracts from leaves (L) and stems (S) of *Rubus caesius* [mg/mL].

Bacteria species	L-W DMSO		L-W water		S-W DMSO		S-W water		L-E DMSO		L-E water		S-E DMSO		S-E water		Ampicillin
	MIC	MBC	MIC	MBC	MIC	MBC	MIC	MBC	MIC	MBC	MIC	MBC	MIC	MBC	MIC	MBC	MIC
*S.* β-hemolytic group G	10	>10	0.63	>5	>10	>10	>5	>5	>10	>10	0.63	>5	>10	>10	>5	> 5	0.016
*M. catarrhalis* PCM2340	2.5	>5	2.5	>10	>5	>5	2.5	>10	2.5	5	0.31	10	2.5	5	0.31	>10	0.0025
*B. subtilis* ATCC6633	5	>5	5	>10	2.5	>5	5	>10	2.5	5	1.25	>5	2.5	5	2.5	>5	0.0004
*S. equinus* ATCC15351	1.25	>5	0.5	>0.5	>5	>5	0.5	>0.5	2.5	>5	0.5	>0.5	2.5	>5	>0.5	>0.5	0.016
*C. diphtheriae*	1.25	>10	0.63	10	5	>10	1.25	>10	0.63	1.25	0.31	2.5	5	10	1.25	10	0.016
*S. epidermidis* ATCC14990	5	>10	10	10	>10	>10	0.04	>10	5	5	0.31	5	>5	10	0.63	5	0.0003
*C. acnes* ATCC6919	1.25	>5	0.5	>0.5	>5	>5	>0.5	>0.5	2.5	>5	>0.5	>0.5	5	>5	>0.5	>0.5	0.032

Extracts were dissolved in water or DMSO. Ampicillin was used as a standard. MIC, minimal inhibitory concentration; MBC, minimal bactericidal concentration.

*S*. β-hemolytic group G, *Streptococcus* β hemolytic group G, *M. catarrhalis*, *Moraxella catarrhalis*, *B. subtilis*, *Bacillus subtilis*, *S. equinus*, *Streptococcus equinus*, *C. diphtheriae*, *Corynebacterium diphtheriae*, *S. epidermidis*, *Staphylococcus epidermidis*, *C. acnes*, *Cutibacterium acnes* (*Propionibacterium acnes*).

**Table 3.** Antifungal properties of water (W) and ethanol (E) extracts from leaves (L) and stems (S) of *Rubus caesius* [mg/mL].

Fungal species	L-W DMSO		L-W water		S-W DMSO		S-W water		L-E DMSO		L-E water		S-E DMSO		S-E water		Amph. B
	MIC	MBC	MIC	MBC	MIC	MBC	MIC	MBC	MIC	MBC	MIC	MBC	MIC	MBC	MIC	MBC	MIC
*C. albicans* ATCC10231	10	10	>10	>10	>10	>10	>10	>10	10	>10	>10	>10	10	>10	>10	>10	0.0005
*C. albicans* ATCC26790	10	>10	50	>50	10	>10	50	>50	>10	>10	50	>50	>10	>10	50	>50	0.001
*C. glabrata* ATCC2001	10	>10	10	>50	5	>10	10	>50	10	>10	10	>50	10	>10	5	>50	0.0003
*C. krusei* ATCC6258	10	>10	10	>50	10	>10	5	>50	10	>10	50	>50	10	>10	50	>50	0.002
*C. parapsilosis* ATCC22019	10	>10	50	>50	10	>10	50	>50	>10	>10	50	>50	>10	>10	50	>50	0.002

Extracts were dissolved in water or DMSO.

Amphotericin B was used as a standard. MIC, minimal inhibitory concentration; MBC, minimal bactericidal concentration.

*C. albicans*, *Candida albicans*; *C. glabrata*, *Candida glabrata; C. krusei*, *Candida krusei*; *C. parapsilosis*, *Candida parapsilosis*; Amph. B, Amphotericin B.

None of the tested extracts showed antimicrobial activity lower than or equal to the references. The water extracts presented the same or weaker activity against the tested bacterial strains than the ethanol extracts. The exception in antibacterial analysis was MIC of L-W extract dissolved in DMSO against *S. equinus* and *C. acnes* (1.25 mg/mL for both strains). DMSO used as a dissolvent for the extracts did not elevate antimicrobial activity of the extracts.

The highest antimicrobial activity was obtained for ethanol extracts from leaves (L-E) dissolved in water against bacteria: *S.* β-hemolytic group G, *S. equinus*, *M. catarrhalis*, *C. diphtheriae, S. epidermidis* (MIC from 0.31 to 0.63 mg/mL). Extracts from stems (S-W and S-E) dissolved in DMSO turned out to be least effective, while dissolved in water strongly acted only on *S. equinus* (MIC 0.5 mg/mL for S-W), *S. epidermidis* (MIC 0.04 mg/mL for S-W), *M. catarrhalis* (MIC 0.31 mg/mL for S-E), and *S. epidermidis* (0.63 mg/mL for S-E). In turn, L-W extract dissolved in water exerted strong antimicrobial activity on *S.* β-hemolytic group G (0.63 mg/mL), *S. equinus* (0.5 mg/mL), and *C. diphtheriae* (0.63 mg/mL).

For other microorganisms, the MIC and MBC values were much higher indicating weak activity of *Rubus caesius* extracts. The tested extracts showed low activity against the reference species of the *Candida* genus (MIC in the range of 10–50 mg/mL). Only *C. glabrata* and *C. krusei* were slightly more sensitive in the presence of S-W and S-E extracts (MIC in the range of 5–10 mg/mL).

For further analysis, ethanol extracts from leaves and stems of *Rubus caesius* were selected due to their high biological activity in previous tests.

### HPLC analysis

**Figure 2.**
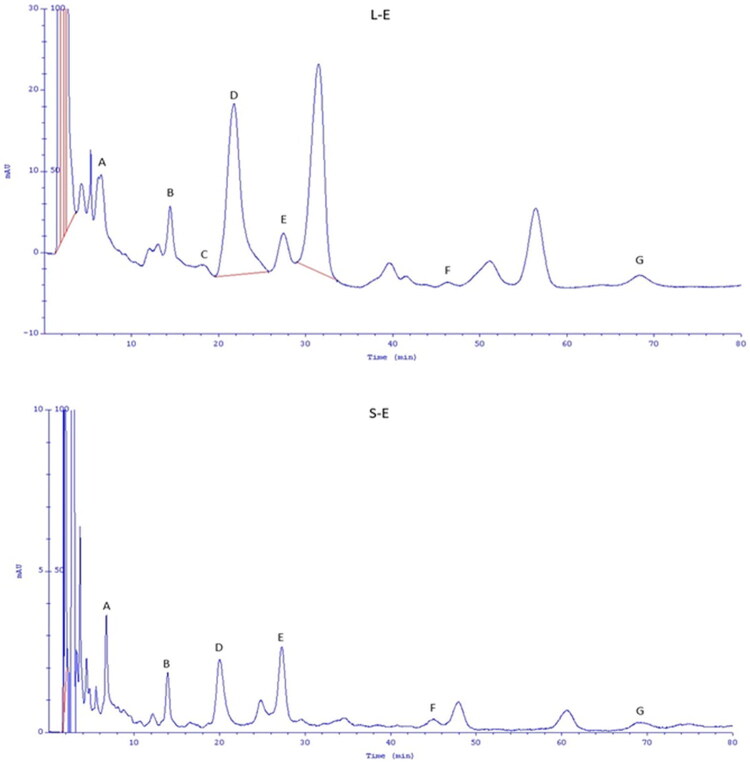
Chromatogram of phenolic acid identified in *Rubus caesius* ethanol extracts from leaves (L-E) and stems (S-E). (A) Gallic acid; (B) protocatechuic acid; (C) caffeic acid; (D) neochlorogenic acid; (E) coumaric acid, (F) vanillic acid and (G) chlorogenic acid. The samples were diluted twenty times before HPLC analysis.

For further phytochemical analysis, ethanol extracts from leaves and stems were selected. The content of selected phenolic acids in L-E and S-E extracts is presented in Table 4 and Figure 2. The following phenolic acids were found: gallic acid, protocatechuic acid, caffeic acid, neochlorogenic acid, coumaric acid, vanillic acid, and chlorogenic acid. The content of phenolic acids varies depending on the morphological parts of the plant. A significantly higher content of these compounds was found in extracts prepared from *Rubus caesius* leaves compared to extracts prepared from stems. In L-E extract, neochlorogenic acid was the most abundant, followed by chlorogenic acid, gallic acid, and protocatechuic acid (Table 4).

**Table 4.** Values of phenolic acids (µg/mL) in ethanol extracts from leaves (L-E) and stems (S-E) of *Rubus caesius.*

Phenolic acid	L-E	S-E
Gallic acid	73.27 ± 2.37^a^	22.20 ± 1.7^b^
Protocatechuic acid	72.16 ± 5.57^a^	27.51 ± 3.26^b^
Caffeic acid	22.08 ± 3.75^a^	n.d.
Neochlorogenic acid	118.01 ± 7.66^a^	29.54 ± 1.21^b^
Coumaric acid	41.05 ± 2.82^a^	16.28 ± 0.31^b^
Vanillic acid	23.24 ± 2.40^a^	4.26 ± 1.18^b^
Chlorogenic acid	98.57 ± 16.13^a^	18.92 ± 3.42^b^

Mean (±standard deviation), (*n* = 3); letters a–b indicate significant differences between the extracts; α = 0.05, n.d., no detected.

#### Permeation skin of hydrogels

Before starting the permeation tests in the Franz diffusion cell, stability tests of the prepared hydrogels were performed. After preparing the hydrogels, their stability and separation was tested. All *Rubus caesius* extract-containing hydrogels maintained their consistency and appearance throughout the test.

**Figure 3.**
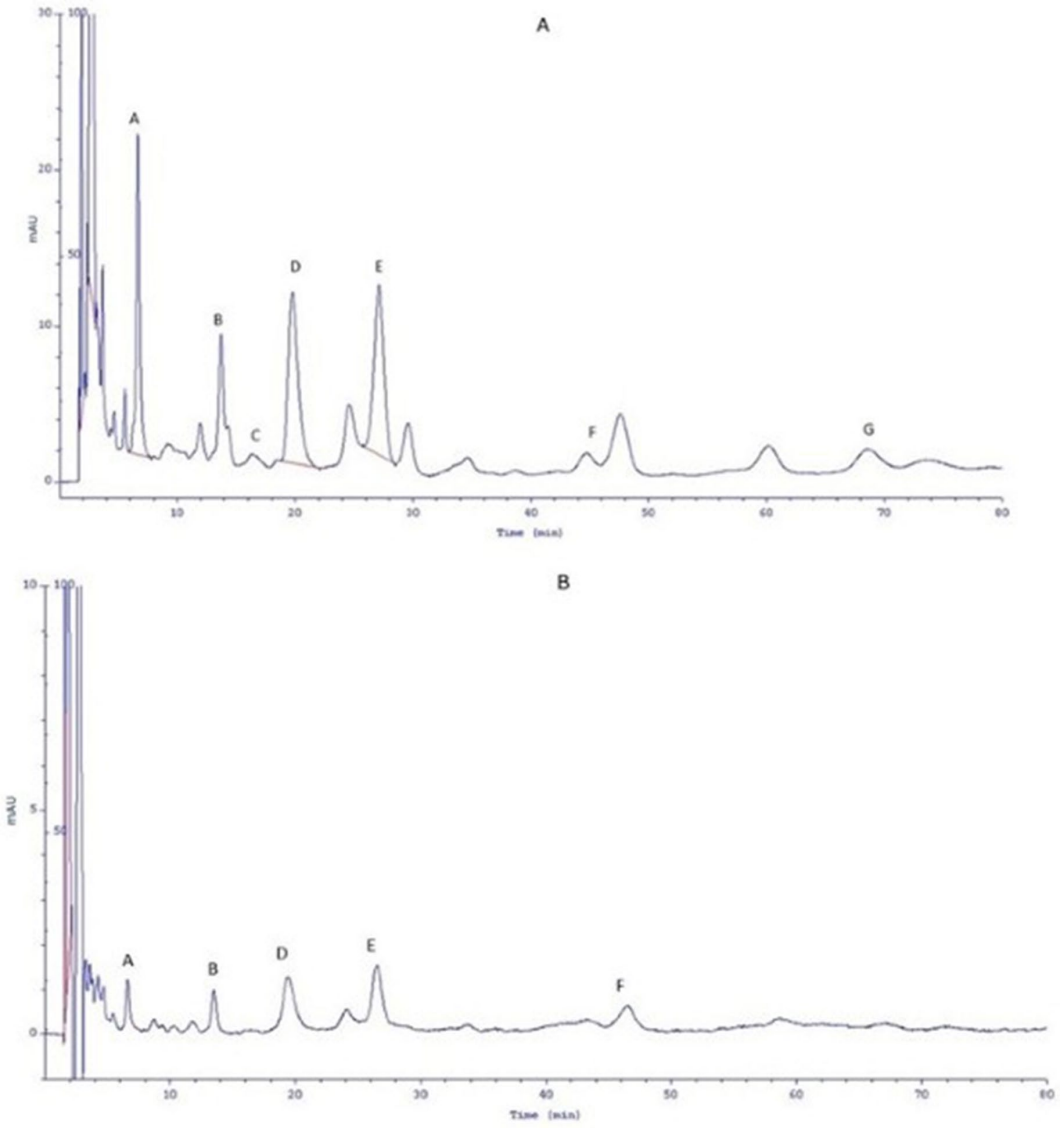
The example chromatograms of the acceptor fluid after 24 h of permeation of phenolic acids from the analyzed hydrogels. (a) Hydrogel *Rubus caesius* leaf extract, (b) hydrogel *Rubus caesius* stem extract. (A) gallic acid; (B) protocatechuic acid; (C) caffeic acid; (D) neochlorogenic acid; (E) coumaric acid; (F) vanillic acid and (G) chlorogenic acid.

The results of the permeation of phenolic acids from hydrogels during the 24-h experiment are shown in Table 5. Whereas Figure 3 shows chromatograms of the acceptor fluid after 24 h of penetration. It was observed that phenolic acids from HL-E penetrate into the skin much better, as well as permeate through the skin. In the case of HL-E, gallic acid and neochlorogenic acid permeated through the skin already in the first hour after application. However, caffeic acid, coumaric acid, vanillic acid, and chlorogenic acid were identified only in the third hour after HL-E application. The highest penetration was observed for neochlorogenic acid, gallic acid, and protocatechuic acid released from HL-E, the cumulative mass of which was collected after 24 h of penetration. The values were 52.00 ± 2.20, 49.62 ± 2.18, and 37.95 ± 2.96 µg·cm^−2^, respectively, which was statistically significant compared to HS-E (Table 5). In general, skin permeation of phenolic acids from HS-E was very low, with some compounds, such as caffeic acid and chlorogenic acid, not permeating at all during the 24-hour study.

**Table 5.** Phenolic acids concentration in the acceptor fluid during 24-hour permeation study after application on the skin HL-E and HS-E.

Time (h)	Gallic acid	Protocatechuic acid	Caffeic acid	Neochlorogenic acid	Coumaric acid	Vanillic acid	Chlorogenic acid
**HL-E (µg/cm^2^)**							
1	3.64 ± 0.12^a^	n.d.	n.d.	0.72 ± 0.17^a^	n.d.	n.d.	n.d.
2	4.54 ± 0.47^a^	3.28 ± 0.31^a^	n.d.	1.21 ± 0.12^a^	n.d.	n.d.	n.d.
3	6.18 ± 0.35^a^	5.86 ± 0.28^a^	2.58 ± 0.20^a^	2.94 ± 0.32^a^	4.97 ± 0.09^a^	0.49 ± 0.25^a^	1.81 ± 0.30^a^
5	10.24 ± 0.97^a^	8.59 ± 0.65^a^	2.84 ± 0.07^a^	7.86 ± 0.40^a^	7.04 ± 0.75^a^	1.76 ± 0.82^a^	3.22 ± 0.69^a^
8	16.09 ± 1.19^a^	11.94 ± 1.91^a^	3.30 ± 0.13^a^	11.47 ± 1.71^a^	8.95 ± 1.00^a^	3.92 ± 1.06^a^	5.35 ± 0.76^a^
24	49.62 ± 2.18^a^	37.95 ± 2.96^a^	7.83 ± 0.83^a^	52.00 ± 2.20^a^	12.45 ± 0.26^a^	15.61 ± 2.42^a^	19.85 ± 2.23^a^
**HS-E (µg/cm^2^)**							
1	n.d.	n.d.	n.d.	n.d.	n.d.	n.d.	n.d.
2	n.d.	n.d.	n.d.	n.d.	n.d.	n.d.	n.d.
3	n.d.	n.d.	n.d.	n.d.	n.d.	n.d.	n.d.
5	3.95 ± 0.18^b^	3.04 ± 0.03^a^	n.d.	n.d.	n.d.	n.d.	n.d.
8	4.69 ± 0.19^b^	3.84 ± 0.18^b^	n.d.	0.21 ± 0.17^b^	5.10 ± 0.11^b^	n.d.	n.d.
24	7.19 ± 0.47^b^	4.57 ± 0.30^b^	n.d	1.21 ± 0.14^b^	5.93 ± 0.08^b^	0.95 ± 0.15^b^	n.d.

Mean (±standard deviation), (*n* = 3); different letters indicate significant differences between the tested extracts; α = 0.05, HL-E, hydrogels with *Rubus caesius* leaf ethanolic extract, HS-E, hydrogels with *Rubus caesius* stem ethanolic extract; n.d., no detected in acceptor fluid.

Table 6 presents the skin permeation parameters of analyzed phenolic acids from hydrogels containing *Rubus caesius* extract. In the hydrogel with leaf extract (HL-E), the steady-state flux (J_SS_) was found to be highest for neochlorogenic acid (2.39 ± 0.13 µg/cm^2^·h) and protocatechuic acid (2.10 ± 0.17 µg/cm^2^·h), followed closely by gallic acid (2.08 ±  0.08 µg/cm^2^·h), with vanillic acid exhibiting the lowest flux (0.53 ± 0.03 µg/cm^2^·h). The permeability coefficients (K_P_·10^3^) followed a similar trend, with the highest values recorded for protocatechuic acid (2.47 ± 0.20 cm/h), gallic acid (2.42 ± 0.09 cm/h), and coumaric acid (2.80 ± 0.35 cm/h). The lag time (L_T_) was the shortest for gallic acid (0.16 ± 0.02 h) and protocatechuic acid (0.63 ± 0.08 h), while coumaric acid exhibited the longest lag time (2.80 ± 0.35 h). Regarding diffusion coefficient (D·10^4^), gallic acid (25.73 ± 1.64 cm^2^/h) and coumaric acid (9.27 ± 0.73 cm^2^/h) showed the highest values, while neochlorogenic acid had the lowest diffusion coefficient (1.55 ± 0.41 cm^2^/h). The skin permeation coefficient (K_m_) was highest for neochlorogenic acid (0.56 ± 0.12), followed by vanillic acid (0.37 ± 0.09), and lowest for chlorogenic acid (0.19 ± 0.06) and protocatechuic acid (0.19 ± 0.04), indicating different affinities for the skin matrix. The percentage of applied dose detected in the acceptor phase after 24 h (Q%_24h_) was highest for gallic acid (5.78 ± 0.25%) and lowest for caffeic acid (0.68 ± 0.07%).

**Table 6.** Skin permeation parameters for phenolic acids with HL-E and HS-E.

Phenolic acid	J_SS_, µg/cm^2^·h	K_P_·10^3^, cm/h	L_T_, h	D·10^4^, cm^2^/h	K_m_	Q%_24h_
**HL-E**						
Gallic acid	2.08 ± 0.08	2.42 ± 0.09	0.16 ± 0.02	25.73 ± 1.64	0.05 ± 0.01	5.78 ± 0.25
Protocatechuic acid	2.10 ± 0.17	2.47 ± 0.20	0.63 ± 0.08	6.57 ± 0.77	0.19 ± 0.04	4.49 ± 0.35
Neochlorogenic acid	2.39 ± 0.13	1.73 ± 0.10	2.68 ± 0.48	1.55 ± 0.41	0.56 ± 0.12	3.72 ± 0.16
Coumaric acid	1.35 ± 0.17	2.80 ± 0.35	2.80 ± 0.35	9.27 ± 0.73	0.15 ± 0.02	2.62 ± 0.05
Vanillic acid	0.54 ± 0.03	1.99 ± 0.23	1.55 ± 0.13	2.68 ± 0.19	0.37 ± 0.09	4.77 ± 0.29
Caffeic acid	0.84 ± 0.02	0.72 ± 0.01	1.22 ± 0.09	3.41 ± 0.28	0.11 ± 0.01	0.68 ± 0.07
Chlorogenic acid	1.00 ± 0.10	0.86 ± 0.09	1.83 ± 0.12	2.27 ± 0.58	0.19 ± 0.06	1.92 ± 0.19
**HS-E**						
Gallic acid	0.88 ± 0.04	5.87 ± 0.25	2.08 ± 0.09	2.01 ± 0.09	1.46 ± 0.12	4.78 ± 0.35
Protocatechuic acid	0.73 ± 0.04	3.91 ± 0.20	2.19 ± 0.10	1.91 ± 0.09	1.02 ± 0.10	2.45 ± 0.16
Neochlorogenic acid	0.07 ± 0.01	0.34 ± 0.04	3.99 ± 0.20	1.05 ± 0.19	0.16 ± 0.05	0.68 ± 0.07
Coumaric acid	n.d.	n.d.	n.d.	n.d.	n.d.	5.37 ± 0.07
Vanillic acid	n.d.	n.d.	n.d.	n.d.	n.d.	3.28 ± 0.52
Caffeic acid	n.d.	n.d.	n.d.	n.d.	n.d.	n.d.
Chlorogenic acid	n.d.	n.d.	n.d.	n.d.	n.d.	n.d.

Jss, steady-state flux; K_P_, permeability coefficient; L_T_, lag time; D, diffusion coefficient; K_m_, skin partition coefficient; Q, the percentage of applied dose; n.d., not detected; the mean ± standard deviation SD (*n* = 3).

In contrast, the hydrogel containing the stem extract (HS-E) showed significantly lower permeation parameters for all acids. Among the detected compounds, gallic acid exhibited the highest steady-state flux (0.88 ± 0.04 µg/cm^2^·h), permeability coefficient (5.87 ± 0.25 cm/h), and diffusion coefficient (2.01 ± 0.09 cm^2^/h), while neochlorogenic acid showed the lowest flux (0.07 ± 0.01 µg/cm^2^·h) and permeability (0.34 ± 0.04 cm/h). For coumaric acid, vanillic acid, caffeic acid, and chlorogenic acid, key permeation parameters could not be calculated due to extremely low or undetectable transdermal transport (n.d., not detected), despite detectable values of Q%_24h_ in some cases (Table 6). These results indicate that leaf-derived extracts provide a more efficient vehicle for the transdermal delivery of phenolic acids compared to stem-derived extracts.

**Figure 4.**
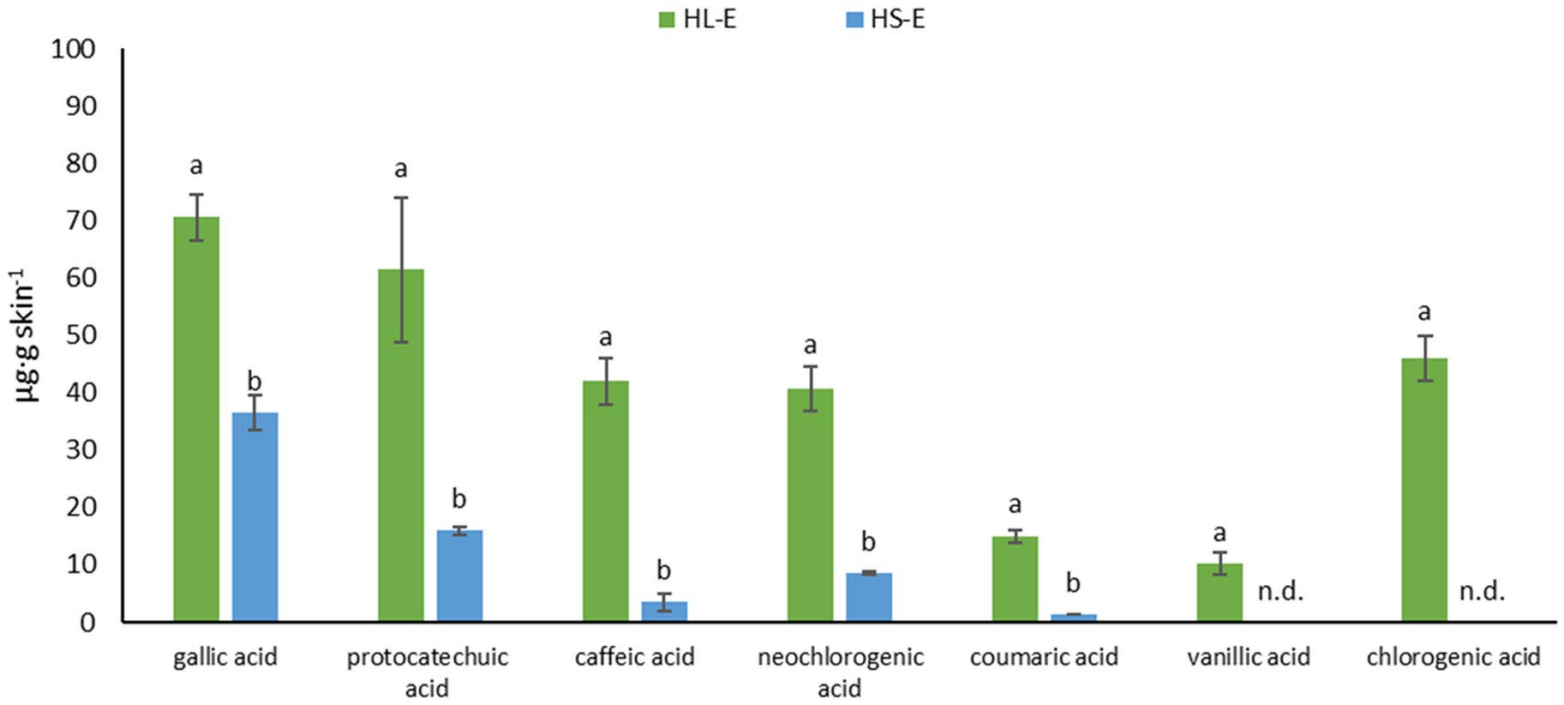
The content of phenolic acids in the solution obtained after the skin extraction collected after 24**-**h penetration. Mean (±standard deviation), (*n* = 3); α = 0.05, HL**-**E – hydrogels with *Rubus caesius* leaf ethanol extract, HS**-**E – hydrogels with *Rubus caesius* stem ethanol extract, n.d., no detected.

Figure 4 shows the accumulation of phenolic acids in the skin. The determination of phenolic acids was performed in the fluid obtained after skin extraction, collected after the end of the 24-h penetration. All analyzed phenolic acids accumulated in the skin. Gallic acid (70.62 ± 4.06 µg/g skin) and protocatechuic acid (61.46 ± 12.57 µg/g skin) accumulated in the skin in the largest amounts after HL-E application. All acids released from HS-E accumulated in much smaller amounts in the skin compared to HL-E (Figure 4).

## Discussion

The skin is highly sensitive to external factors. Exposure to stressors especially air pollution, and strong UV radiation adversely affects skin health. Long-term exposure to such harmful factors can cause permanent changes in skin structure, leading to premature aging.

Various plant materials from the Rosaceae family exhibit high polyphenolic content and diverse biological activities. *Rubus caesius* is rich in anthocyanins, polyphenols, and tannins (Grochowski et al. 2020; Hering et al. 2022). Differences in polyphenolic composition, antioxidant, antimicrobial, and antihyaluronidase activity among extracts obtained from young stems and leaves were described previously (Hering et al. 2022). This study continues our prior research, aiming to identify extracts with the potential to regulate skin condition and inhibit premature aging.

We analyzed extracts from leaves and stems, which are available throughout the vegetation period. Water and ethanol were used as solvents due to their low toxicity and ease of removal. These are important key factors for dermocosmetic applications.

Ethanol extracts from *Rubus caesius* leaves and stems were more effective in scavenging free radicals, with IC_50_ values comparable to those of ascorbic acid. However, as demonstrated in our previous studies, water extracts also exhibited strong antioxidant activity (Hering et al. 2022). In another assay evaluating antioxidant capacity, in this work ethanol extracts again showed a significant advantage, displaying activity much closer to that of ascorbic acid than the water extracts. Detected phenolic acids are known for their strong antioxidant properties, though after oral administration are metabolized, resulting in low bioavailability. Transdermal delivery of those molecules can effectively strengthen the skin’s protective properties against oxidative stress and the destruction of structural macromolecules (Nisa et al. 2024). The highest concentrations of phenolic acids was detected in the ethanol extract from the leaves, which likely accounts for its high activity. Gallic acid and protocatechuic acid, as well as chlorogenic and neochlorogenic acids are known antioxidants, and as their amount in the extracts is high the main antioxidant activity of the extracts might be connected with their presence (Sato et al. 2011; Naveed et al. 2018; Nisa et al. 2024; Xiang et al. 2024). Phenolic acids detected in ethanol extracts of *Rubus caesius* leaves and stems demonstrate, according to the literature, significant antiaging potential. Studies have shown that they not only exhibit free radical scavenging properties but also inhibit the activity of enzymes in the extracellular matrix of the skin. Several Matrix Metalloproteinase (MMP) activity are induced by ROS. As the phenolic acids are capable to capturing free radicals, they are also able to inhibit the activation of enzymes and processes responsible for the progressive skin aging processes. The antityrosinase potential further supports the antioxidant properties of *Rubus caesius* extracts. Tyrosinase activity within skin layers leads to the production of reactive quinones, which can alter the structure and function of macromolecules and trigger a cascade of chemical reactions in the skin (Ito et al. 2020). Overactivation of tyrosinase, the key enzyme in melanin synthesis, can result in unwanted skin pigmentation, inflammation, and even cancer-like changes (Ito et al. 2020). Inhibiting this enzyme may help maintain an even skin tone and reduce oxidation and inflammation associated with quinones. Increased collagenase activity is linked to wrinkle formation, inflammation processes and weakening of skin structures, leading to its sagging (Varani et al. 2006; Reilly and Lozano 2021). Chlorogenic acid and gallic acid have potential to inhibit collagenase and tyrosinase effectively (Kim 2007; Li et al. 2014; Girsang et al. 2024). Caffeic acid and protocatechuic acid in addition to having strong free radical scavenging abilities, inhibit the activity of collagenase and, in higher doses, also tyrosinase (Girsang et al. 2020a; 2020b), while vanillic acid limit strongly collagenase activity and might be responsible as well for tyrosinase inhibition (Jiamphun and Chaiyana 2022). Evaluation of the influence of the tested extracts on the activity of tyrosinase confirmed a significant advantage of inhibiting its activity by *Rubus caesius* ethanol extracts. Additionally, the extract from leaves was as potent as kojic acid. Similar findings were observed in the collagenase inhibition assay: while all extracts showed some activity, the ethanol stem extract demonstrated the highest level of inhibition.

The skin surface is a natural habitat for numerous bacterial strains that interact with each other and influence the skin’s condition and health. Homeostasis of the skin microbiome is essential for proper immune system function and helps prevent the overgrowth of pathogenic strains (Fredricks 2001; Flowers and Grice 2020). In this study, various bacterial and fungal strains were cultured in the presence of *Rubus caesius* extracts dissolved in either water or DMSO. The antimicrobial activity of the extracts was not enhanced by DMSO as a solvent.

The results indicated that *Rubus caesius* extracts did not affect the survival of *Bacillus subtilis* or certain *Candida* strains, which when are in balance, form part of the skin’s natural microbiome (Kühbacher et al. 2017; Tuor and LeibundGut-Landmann 2023). However, even a sudden shift in individual commensal strains can trigger pathological conditions such as allergies or inflammation (Moskovicz et al. 2021). These findings highlight the selectivity of *Rubus caesius* extracts: they do not disturb beneficial skin flora but are effective against highly pathogenic strains, such as *Corynebacterium diphtheriae*, a producer of diphtheria toxin and known for its antibiotic resistance (Cieplewicz et al. 2019)*. Staphylococcus epidermidis* is associated with biofilm formation, a major contributor to transplant rejection (Brown and Horswill 2020; Severn and Horswill 2023). *Moraxella catarrhalis* is known for causing mucosal infections (Karalus and Campagnari 2000), and together with *S. epidermidis*, can lead to deep skin ulcers and ecthyma (Rabionet et al. 2016). These highly pathogenic bacterial strains were found to be sensitive to ethanol extracts of *Rubus caesius*, particularly those obtained from the leaves. The difference in activity between the tested extracts may be due to the varying amounts of phenolic acids. As weak acids, they are able to penetrate the cell membrane and can acidify the cytoplasm, causing among others bacterial death. All identified compounds exhibit antimicrobial activity, though using different metabolic pathways. Lipophilicity and pKa are important factors in their antibacterial activity (Alibi et al. 2021). It has been observed that the number and position of substituent groups on the aromatic ring, the length of the side chain, and the number of unsaturated bonds influence the degree of antimicrobial activity of phenolic acids (Kauffmann and Castro 2023; Kumar and Goel 2019). It was observed that compounds with long alkyl side chains were more active in inhibiting Gram-positive bacteria, while compounds with medium alkyl chains inhibited Gram-negative bacteria (Kauffmann and Castro 2023; Bouarab-Chibane et al. 2019). The lipophilicity of phenolic acids is one of the factors determining antimicrobial activity; the higher the lipophilicity of a compound, the greater its ability to inhibit bacterial growth. It has been shown that as hydroxyl groups are replaced by methoxyl groups, unsaturation occurs in the molecules of compounds, which increases the lipophilicity of acids and decreases the pH, which causes acidification of the plasma membrane of pathogens, improving antibacterial activity (Cueva et al. 2010; Sánchez-Maldonado et al. 2011; Kauffmann and Castro 2023). Among the phenolic acids present in the tested extracts, caffeic acid is the most studied.

Luis et al. proposed that the mechanism of action of caffeic acid is related to cell membrane damage and changes in the oxidative metabolism of *S. aureus* cells (Luís et al. 2014; Miklasińska-Majdanik et al. 2018). Furthermore, caffeic acid, as a phenolic acid, has strong nucleophilic properties, allowing it to donate electron pairs to electrophilic functional groups of proteins and/or lipids of the plasma membrane, likely leading to membrane dysfunction (Nquefack et al. 2004; Hayouni et al. 2008; Miklasińska-Majdanik et al. 2018). Caffeic acid has been found to inhibit the secretion of α-hemolysin in *S. aureus*, a cell membrane-dependent process, and exhibit stronger antimicrobial activity than gallic acid, vanillic acid, and protocatechuic acid (Vaquero et al. 2007; Miklasińska-Majdanik et al. 2018, Luís et al. 2014). The high antibacterial activity of caffeic acid appears to be due to the presence of a propenoic side chain, which reduces its polarity compared to the hydroxybenzoic structure of protocatechuic acid. Furthermore, phenolic acids are thought to exert various mechanisms of action on bacterial cells. For example: intracellular K^+^ ion efflux and membrane permeabilization are known for gallic acid (Borges et al. 2013; Oulahal and Degraeve 2021), chlorogenic and gallic acids lower extracellular pH, while caffeic acid, protocatechuic acid, and vanillic acid have capability to penetration or accumulation of the undissociated form in the cell membrane, while p-coumaric acid has significant antimicrobial activity in the dissociated form (Pernin et al. 2019; Oulahal and Degraeve 2021). Induction of intracellular metabolic imbalance by chlorogenic acid through action on the tricarboxylic acid cycle and glycolysis, leads to metabolic disturbances and death (Wu et al. 2020). Morphological defects have been observed in the polar ends of bacteria treated with gallic or protocatechuic acid. Treatment with vanillic acid, on the other hand, resulted in disruptions in the cell division process by inhibiting formation of the intercellular septum (Alvarado-Martinez et al. 2020; Oulahal and Degraeve 2021). Gallic acid decreased the expression of all genes located in Salmonella Pathogenicity Island 1 (SPI-1), responsible for bacterial pathogenicity. Protocatechuic acid has also proven activity in reducing the expression of key regulatory genes (Alvarado-Martinez et al. 2020; Oulahal and Degraeve 2021). P-coumaric acid inhibited DNA and RNA synthesis, membrane cell interaction, and RecA protein (Borges et al. 2013, De Rossi et al. 2025; Bae et al. 2022; Minich et al. 2022). Gallic acid, vanillic acid, and caffeic acid (Ugurlu et al. 2016; Oulahal and Degraeve 2021) have been shown to inhibit the formation or elimination of biofilms containing undesirable foodborne pathogenic microorganisms (Oulahal and Degraeve 2021).

As *in vitro* tests revealed significantly lower biological activity of the water extracts, ethanol extracts of *Rubus caesius* were selected for further studies *ex vivo*.

The biological activity of skin—applied preparations largely depends on the permeation of key active compounds. For topical applications, these substances must be effectively released from the formulation and reach all skin layers, including the underlying tissues (Bertges et al. 2020; Nowak et al. 2021b). In recent years, there has been growing interest in using plant extracts in various dermatological and cosmetic products (Zagórska-Dziok et al. 2021b). Plants are rich in secondary metabolites with diverse pharmacological effects, including those relevant to skin health. Among these, phenolic acids hold particular importance due to their regenerative potential for both the epidermis and deeper layers.

Phenolic acids are valued for their antioxidant, anti—inflammatory, antiaging, and antibacterial properties (Korkina et al. 2012; Michalak 2022). The assessment of the penetration of these compounds through the skin is an important element in modeling cosmetic preparations containing plant extracts. The penetration degree of these substances may be different, depending on the compound’s physical and chemical properties, such as lipophilicity, molecular structure, and polarity (Alonso et al. 2014; Bertges et al. 2020; Nowak et al. 2021a). To exert their beneficial effects, phenolic acids must be released from the topical preparation and successfully permeate the stratum corneum (SC), the primary barrier of the skin (Zillich et al. 2013; Murphy et al. 2022).

The next aim of our research was to evaluate the penetration of selected phenolic acids from two hydrogels containing dry ethanol extracts from the leaves (HL-E) and stems (HS-E) of *Rubus caesius*. The penetration test was conducted using a Franz diffusion cell with pig skin, which is structurally similar to human skin (Jacobi et al. 2007).

The phenolic acids penetrated the skin to varying degrees. It was observed that certain phenolic acids permeated the skin more effectively from HL-E than from HS-E. In the case of HL-E, some acids—such as gallic acid and neochlorogenic acid—were detected in the receptor fluid as early as the first hour post-application. The highest permeation values were recorded for gallic acid, vanillic acid, protocatechuic acid, and neochlorogenic acid from HL-E, at 5.78%, 4.77%, 4.49%, and 3.72%, respectively. Notably, neochlorogenic acid showed significantly lower permeability from HS-E. Probably, its lower content in S-E compared to L-E had a significant impact for the result. As we know, plant extracts contain a whole pool of various secondary metabolites, which, depending on their concentration, may interact synergistically, which may also increase the permeation of some compounds (Hering et al. 2021). In addition, the leaf probably contains other substances not found in the stem that may demonstrate this effect. Generally, in our study, the leaf extract hydrogel outperformed the stem extract hydrogel in promoting permeation, as evidenced by higher JSS, KP, and D values. Gallic acid consistently demonstrated superior permeation characteristics in both extracts. These findings highlight the importance of selecting the appropriate *Rubus caesius* extract and active compound when designing formulations for dermal application. The results suggest that the leaf extract hydrogel is more effective in promoting skin permeation, with gallic acid standing out as a compound with particularly strong permeation potential.

Previous authors also reported rapid skin penetration of gallic acid from microemulsions containing *Glochidion wallichianum* extract and with *E. angustifolium* ethanol extract (Nowak et al. 2021a; Sae and Sakdiset 2020).

The vehicle used plays a key role in the release and delivery of active substances into the skin. In our study, hydrogel was selected due to its simple composition, high water content, and biocompatibility. Hydrogels containing plant extracts are gaining increasing importance in cosmetology, medicine, and pharmacy. These formulations, enriched with a variety of bioactive compounds, can exert multiple effects, including antioxidant, antibacterial, and anti-inflammatory activities (Zagórska-Dziok and Sobczak 2020; Gavan et al. 2022; Zagórska-Dziok et al. 2023). Previous studies have shown successful therapeutic applications of hydrogels enriched with natural extracts. For instance, a carbomer-based hydrogel containing 2% ethanol extract of *Punica granatum* peels contributed to healing a chronic leg ulcer in a 76-year-old patient (Fleck et al. 2016). Hydrogel films composed of agarose, κ-carrageenan, and glycerol, enriched with an aqueous extract from *Cryphaea heteromalla*, exhibited notable antioxidant activity (Ditta et al. 2020). Similarly, hydrogels containing 5% *Epilobium angustifolium* extract demonstrated anti-inflammatory and antioxidant properties and promoted wound healing *in vitro* (Nowak et al. 2022). Our own research supports these findings, indicating that hydrogels containing *Rubus caesius* leaf extract not only ensured consistent delivery of phenolic acids but also promoted their accumulation and transdermal permeation, validating their suitability as carriers in cosmetic and dermocosmetic formulations. Hydrogels also offer several advantageous properties, such as biodegradability, biocompatibility, nontoxicity, and bifunctionality (Zagórska-Dziok and Sobczak 2020; Michalak et al. 2021; Nowak et al. 2022). Our previous research demonstrated that phenolic acids showed significantly better skin penetration when delivered *via* hydrogel compared to an emulsion (Nowak et al. 2021b). Similarly, Žilius et al. reported the highest penetration of phenolic acids—such as coumaric, caffeic, and ferulic acids—from a hydrogel containing propolis extract. These authors suggested that vehicles with lower oil content promote faster release rates of phenolic compounds, while the higher viscosity of emulsions, due to their oil phase, impedes the diffusion of these active molecules (Žilius et al. 2016).

Preformulation testing, such as the stability of cosmetic preparations, is important for ensuring the appropriate quality of preparations used. All *Rubus caesius* extract-containing hydrogels maintained their consistency and appearance throughout the test. Active substances must penetrate the SC to reach cells located in the deeper layers of the epidermis, the dermis, or even the cutaneous microcirculation. In some analgesic drugs, rapid penetration is preferred to achieve a quick therapeutic effect. In contrast, for plant-derived compounds such as phenolic acids, greater accumulation within the skin is desirable, where they can exert antioxidant and antiaging effects (Bertges et al. 2020; Nowak et al. 2022; Ossowicz-Rupniewska et al. 2022).

For this reason, ethanol extracts from *Rubus caesius* leaves show the highest potential for use in cosmetic and dermocosmetic applications. The active compounds demonstrated the ability to be released from the hydrogel, penetrate the SC barrier, accumulate in the skin, and even permeate through it. Compounds such as gallic acid, neochlorogenic acid, chlorogenic acid, protocatechuic acid, and caffeic acid can act within the viable skin layers—neutralizing free radicals, inhibiting enzymes responsible for the degradation of ECM macromolecules, and limiting bacterial infections—without disrupting the natural skin microbiome.

Ethanol stem extracts, when incorporated into hydrogels, may be more suitable for dermocosmetic products targeting the skin surface. Since phenolic acids in these extracts did not effectively penetrate the SC, they may serve primarily to protect the skin from ROS and pathogenic bacterial imbalance. Water extracts also hold some promise in dermocosmetic use; however, their antimicrobial, antioxidant, and enzyme-inhibitory effects were significantly lower than those of the ethanol extracts.

This results, suggests promising potential for the use of European dewberry leaf extract in cosmetic and dermocosmetic formulations. And therefore, further analyses including dermoprotective effects and safety assessments through *in vivo* studies are recommended especially for ethanol extracts obtained from *Rubus caesius* leaves.

## Conclusions

Ethanol extracts derived from *Rubus caesius* leaves collected during the spring vegetation season represent a valuable source of phenolic acids capable of protecting the skin against oxidative stress and pathogenic bacteria. Additionally, these extracts show potential in safeguarding macromolecules in the deeper layers of the skin, particularly collagen, and in preventing the overexpression of tyrosinase, which can lead to irregular melanin distribution. Owing to these properties, the extract may serve as a promising ingredient in dermocosmetic formulations and warrants further *in vivo* investigation.

## Data Availability

The data presented or analyzed in this study are available from the corresponding author upon reasonable request.
